# Supplementary material for: A Cross-Sectional Study of SARS-CoV-2 Antibodies and Risk Factors for Seropositivity in Staff in Day Care Facilities and Preschools in Denmark
Source: Microbiol Spectr. 2022 Dec 22;11(1):e04174-22. doi: 10.1128/spectrum.04174-22 (PMC9927135; doi:10.1128/spectrum.04174-22)
Supplement: Supplemental file 1 — Fig. S1. Download spectrum.04174-22-s0001.pdf, PDF file, 0.1 MB [file spectrum.04174-22-s0001.pdf]

Supplementary figure 1: General national recommendations from the Danish Health authorities during the study period

1	Keep a distance of 2 meters whenever possible (at least 1 meter).
2	Avoid handshakes, hugging and kissing on the cheek.
3	If you get COVID-19 symptoms, isolate yourself and have an antigen and/or PCR test performed.
4	On a regular basis, ventilate your home by opening windows and doors.
5	Avoid gathering too many people indoors.
6	Sneeze or cough into your sleeve.
7	Use hand sanitizer or frequent hand washing.
8	Clean thoroughly and on a regular basis, especially surfaces that are touched frequently.
9	Persons aged 12 and up are required to wear face masks or shields in the following places: public transportation, wholesale and retail stores, premises for cultural, sports, and club activities, religious communities, day-care facilities, schools, education institutions, public and private hospitals and clinics, and premises where clients are offered services involving physical contacts.
